# Attitudes of Christian leaders and congregants in South Africa towards mental illness and the mentally ill

**DOI:** 10.4102/sajpsychiatry.v31i0.2399

**Published:** 2025-04-09

**Authors:** Zamahlubi T. Dlamini, Vinola Poliah, Navanthree Govender

**Affiliations:** 1 Department of Psychiatry, Faculty of Health Sciences, University of the Witwatersrand, Johannesburg, South Africa

**Keywords:** mental illness, stigma, Christian community, church, attitudes, congregants

## Abstract

**Background:**

Mental illness and substance use disorders significantly contribute to the global disease burden, and limited access to mental health services exacerbates this problem. Initially, many individuals seek help from religious leaders and traditional healers. Given that 80% of South Africa’s (SA) population identifies as Christian, churches may influence mental health help-seeking behaviour.

**Aim:**

This study aimed to determine the attitudes of Christian leaders and congregants towards the mentally ill using the Community Attitudes Towards the Mentally Ill (CAMI) scale.

**Setting:**

The study was conducted in Soweto, a diverse peri-urban settlement in Johannesburg.

**Methods:**

This was a cross-sectional survey where participants completed a demographics questionnaire and the CAMI scale, which measures attitudes across four sub-scales: Authoritarianism (AU), Benevolence (BE), Social Restrictiveness (SR), and Community Mental Health Ideology (CMHI). Low AU and SR scores and high BE and CMHI scores indicated low stigma towards mental illness.

**Results:**

There were 51 participants, predominantly female (80.4%), aged 25–35 years (58.8%) and possessing tertiary education (82.4%). No significant differences emerged between leaders and congregants. Participants with a personal history of mental illness exhibited more positive attitudes, while familiarity with affected individuals did not significantly influence attitudes.

**Conclusion:**

The study highlights the importance of collaboration between mental healthcare providers and the Christian community in South Africa, emphasising the need for cross-denominational engagement and further research to improve culturally relevant mental healthcare.

**Contribution:**

These findings underscore the church’s potential role in promoting mental health support.

## Introduction

Mental illness causes a significant burden of disease accounting for 7.4% of disease burden worldwide.^1^ Mental and substance use disorders are the leading global cause of all non-fatal burden of disease and are also classified as the fifth leading disorders globally that cause overall disease burden. These figures have risen significantly since 1990.^1^

Limited access to mental health services leads to prolonged untreated symptoms, which negatively affects the course and outcome of the disease.^2^ Barriers to accessing mental health services include difficulty recognising mental illness, perceived stigma, affordability, awareness of the availability of services and a shortage of facilities and personnel.^3^

In South Africa (SA), these challenges are compounded by the shortage of psychiatrists and mental health infrastructure.^4^ South Africa also has a high prevalence of human immunodeficiency virus (HIV), trauma, substance use, unemployment, violence and poverty, all of which increase the risk of mental illness and disability in the community.^5,6,7,8^ This increased burden of disease coupled with the under-resourced infrastructure at all levels of care means that medical pathways for seeking treatment for mental illness may be difficult to access and may not cater to every person in need of the services.

It is for this reason, among others, that a significant portion of the population globally access services in churches, including seeking guidance and assistance concerning mental health issues.^9^ People with mental health problems are more inclined to seek help from religious leaders and traditional healers first, who, more often than not, do not refer patients to health facilities.^10,11^

Approximately 80% of SA’s population identifies as Christian with the largest group, 40.8%, belonging to the Africa Independent denomination (various Zion Christian Church and Zionist churches), followed by mainstream denominations (39.8%), other Christian groups (11.9%) and Pentecostal or Charismatic (7.3%).^12^

Members of the Christian community often have high regard for their leaders and take the advice and counsel offered to them.^9^ The church plays a significant role in influencing the help-seeking behaviour of church members, including those with mental illness.^13^ They can also influence how congregants view mental illness as well as offer support to them.^14^ South Africa’s large Christian population may suggest a substantial representation of Christians within psychiatric settings. It is crucial to understand the attitudes of the Christian community towards mental illness and the mentally ill, as these attitudes, defined as a mental view that influences behaviour,^15^ play a crucial role. These attitudes may affect the experiences that people seeking help from the church have and contribute to not only their personal view of mental illness and the mentally ill but also determine if they access mental health care services.

Literature has highlighted how misinformation surrounding mental illness can be linked to stigma, which further perpetuates under-diagnosis and under-treatment in South African communities.^16^ Literature also demonstrated the need for continuous education around mental illness, in particular the neurochemistry and the importance of appropriate pharmacological and psychological management.

Several studies in Africa have been conducted describing people’s attitudes towards mental illness focusing on the general community.^16,17,18,19^ Some international studies have explored attitudes among Christian leaders and congregants.^14,20,21,22,23^ while only two South African studies focused specifically on the Christian community.^24,25^ While various studies have explored attitudes towards mental illness, there remains a gap in understanding the specific perceptions within the South African Christian community.

There are a few factors other than religious affiliation that affect the attitudes of the general public towards people with mental illness. These include level of education, whether they reside in a rural or urban area, age, exposure to people living with a mental illness, as well as perceived causes of mental illness.^26^ The general community has been shown to believe that mental illnesses have a spiritual connotation. The best treatment for these individuals is therefore thought to be received from religious and traditional healers.^21^

Studies have been conducted to explore clergy attitudes towards mental illness. In Benin City in Nigeria, 70% of participants believed it was easy to differentiate mentally ill individuals from the general population because of distinctive characteristics, while nearly half attributed mental illness to personal failing, specifically a lack of discipline.^21^ In Lebanon, clergy from various Christian denominations were surveyed about their knowledge, attitudes and beliefs regarding mental health. Despite 81.6% of respondents having attended psychology courses, there remained a prevalent stigma and discrimination towards individuals with mental illness.^20^

The attitudes of Christian leaders and the Christian community contribute to beliefs about mental illness, as well as to the support rendered by the church to the mentally ill. Negative attitudes of Christian leaders towards mental illness affect their members as shown in an online study conducted in the United States (US).^14^ Additionally, a study found that Christian leaders who attributed mental illness to spiritual causes were more likely to dismiss medical diagnoses.^26^

A study on congregants in Ghana demonstrated that accurate information that dispels stigma can improve help-seeking behaviour in the church context. The findings showed that in addition to spiritual causes, the participants were able to identify that other factors can cause mental illness like substance use, life stressors and trauma. This belief in a multi-factorial cause translated into help-seeking behaviour where both medical and spiritual treatments were advised.^23^

If religious leaders can identify mental illness, then they can refer appropriately and timeously, aiding in the adequate management of the psychiatric illness. This was demonstrated in a study conducted in Texas, US on Baptist pastors. The results showed that the pastors recognised a biological cause of mental illness and were referring the congregants who needed help to mental health services.^22^

From the literature, it is evident that both the general community and clergy may have mixed attitudes towards mental illness and the mentally ill. Negative attitudes, however, may impede the help-seeking behaviours of those with mental illness, influence the encounters they have with the church and may delay psychiatric intervention.

### Aim

This study aimed to determine the attitudes of Christian leaders and congregants in Soweto, Johannesburg towards the mentally ill.

### Objectives

This study had the following objectives:

- To describe the demographic profile of church leaders and congregants who participated in the study.
- To determine the attitudes of these church leaders and congregants toward the mentally ill, using the Community Attitudes Towards the Mentally Ill (CAMI) scale.^24^

## Methodology

### Study design

This was a cross-sectional study design.

### Method

Participants, that is Christian leaders and members of identified churches in Soweto, were given two questionnaires to fill out, one for demographic information and another for the CAMI scale.

### Study setting

The study was conducted in Soweto, a town in the city of Johannesburg. Soweto is a peri-urban settlement in the south-west of Johannesburg with a diverse population group and people across all economic sectors. It was chosen as the area of study because of its accessibility and diverse Christian population.

### Study population and sampling

The study population included all adults who identify as Christian and are members or leaders belonging to the African Independent, Mainstream, Pentecostal or Charismatic and other Christian denominations. Church leaders were approached in person and informed of the study, and assistance was requested to avail the questionnaires to the congregants. Although questionnaires were made available both in paper-based and online formats, all participants who agreed, completed online questionnaires. Efforts to improve response rates included follow-ups through church leaders and reassurances regarding confidentiality and anonymity.

Inclusion criteria:

- Adult female and male church members who are part of the leadership or are congregants of one of the above Christian churches.
- A fair level of English proficiency and literacy.

Exclusion criteria:

- Church members below the age of 18 years.
- Visitors or non-members of the selected churches.
- Members with inadequate English proficiency and literacy.

### Sample size

A voluntary purposive sampling method was used to select participants. A total of 51 participants completed the questionnaires. A minimum sample size of 166 was determined to be necessary for achieving statistical significance. The sample size was calculated using the formula (Equation 1):


n=Z2pq/e2 [Eqn 1]

where *e* is the desired level of precision (i.e. the margin of error), *p* is the (estimated) proportion of the population which has the attribute in question and *q* is 1 – *p*. If *Z* = 1.96, and *p* is set to 0.33 and the *q* value set at 5%, a sample size of 166 is generated.

### Measuring tool

The CAMI scale was developed to measure the general public’s attitudes towards mental illness by Canadian researchers.^27^ It is a self-administered questionnaire and has four sub-scales each with 10 items. It rates the 40 items on a Likert scale (1 – strongly agree to 5 – strongly disagree). Participants are requested to answer questions regarding their beliefs about mental illness and people living with mental illness. The responses to individual items are added together to form a subscale score ranging from 10 to 50 on each subscale. The subscales are Authoritarianism (AU) which refers to one’s view of individuals with a mental illness as inferior and requiring supervision. It measures the participant’s way of looking at the disease and knowledge of how it develops and how people living with mental illness should be taken care of. Benevolence (BE) refers to a humanistic and sympathetic view of people living with a mental illness. Social Restrictiveness (SR) corresponds to the belief that people with a mental illness are a danger to society, and Community Mental Health Ideology (CMHI) refers to the acceptance of mental healthcare services and the integration of mental healthcare users into the community. Higher scores reflect greater agreement with the concept of each sub-scale. Participants who are considered tolerant would be expected to have higher scores on the sub-scales of BE and CMHI while scoring lower on the AU and SR sub-scales.^28^ The CAMI scale was found to be reliable and valid by various studies, including studies conducted in Africa.^17,19^ The developers gave permission on their website for the scale to be used for research, educational and academic purposes.^27,29^

### Data collection

A standardised CAMI questionnaire and a demographics questionnaire were used to look at the participants’ age, gender, employment status, level of education, personal experience with mental illness, denomination and position in the church.

A link was made available to church leaders and congregants to complete.

### Data analysis

The data for this study were collected using Microsoft Excel™. Statistical analyses were conducted in R software (version 4.00; www.R-project.org). Shapiro-Wilk’s tests indicated that all CAMI category scores were normally distributed, so data were analysed using parametric tests. Linear regressions were used to compare the association between the four CAMI categories (AU, BE, CMHI and SR). Welch’s *t*-tests were used to analyse the effects of congregant versus leader, and personal history of mental illness on each CAMI category. All tests were two-tailed, and model significance was set at 0.05. Categorical data are reported descriptively as counts and parentages and continuous data as mean and standard deviation. Data are presented as charts, tables or in text.

### Ethical considerations

Approval for the study was sought from the University of the Witwatersrand Human Resources Ethics Committee (Medical), and ethics consent was received on 10 August 2023, with Clearance Certificate Number M230542 M230810-B-0002 before data collection commenced.

Permission to conduct research was also obtained from the church leadership in the selected churches. Written consent was obtained from all participants before completion of the questionnaire. All participants remained anonymous so that confidentiality was always maintained. The names of individual churches were not stated.

## Results

A total of 51 churchgoers participated in this study. They were predominantly female (*n* = 41, 80.4%), aged between 25 years and 35 years (*n* = 30, 58.8%), possessed a tertiary-level education (*n* = 42, 82.4%) and were primarily affiliated with the Pentecostal or Charismatic denomination (*n* = 27, 52.9%) (Table 1). Congregants (*n* = 29) outnumbered leaders (*n* = 22) slightly. While a greater proportion reported no personal history of mental illness (*n* = 36, 70.6%), a larger number acknowledged knowing someone with mental illness (*n* = 39, 76.5%) (Table 1).

**TABLE 1:** Socio-demographic and other characteristics of congregants and leaders in this study.

Variable	Congregant (*N* = 29)		Leader (*N* = 22)	
	*n*	%	*n*	%
**Gender**				
Female	25	86.2	16	72.7
Male	4	13.8	6	27.3
**Age (years)**				
18–25	6	20.7	5	22.7
25–35	18	62.1	12	54.5
35–40	3	10.3	1	4.5
40–50	2	6.9	4	18.2
**Level of education**				
High school	8	27.6	1	4.5
Tertiary	21	72.4	21	95.5
**Denomination**				
African Independent	3	10.3	0	0.0
Mainstream	6	20.7	2	9.1
Pentecostal or Charismatic	11	37.9	16	72.7
Other	8	27.6	3	13.6
Not specified	1	3.4	0	0.0
Unsure	0	0.0	1	4.5
**Personal history of mental illness**				
Yes	12	41.4	3	13.6
No	17	58.6	19	86.4
**Knew someone with a mental illness**				
Yes	24	82.8	15	68.2
No	5	17.2	7	31.8

The mean and standard deviation scores for the CAMI categories were as follows: AU = 24.73 (5.18), BE = 41.61 (4.65), SR = 21.43 (5.47) and CMHI = 40.14 (4.44). The low AU, high BE, low SR and high CMHI mean scores indicate that the participants viewed mental health to be of low stigma.

**FIGURE 1:**
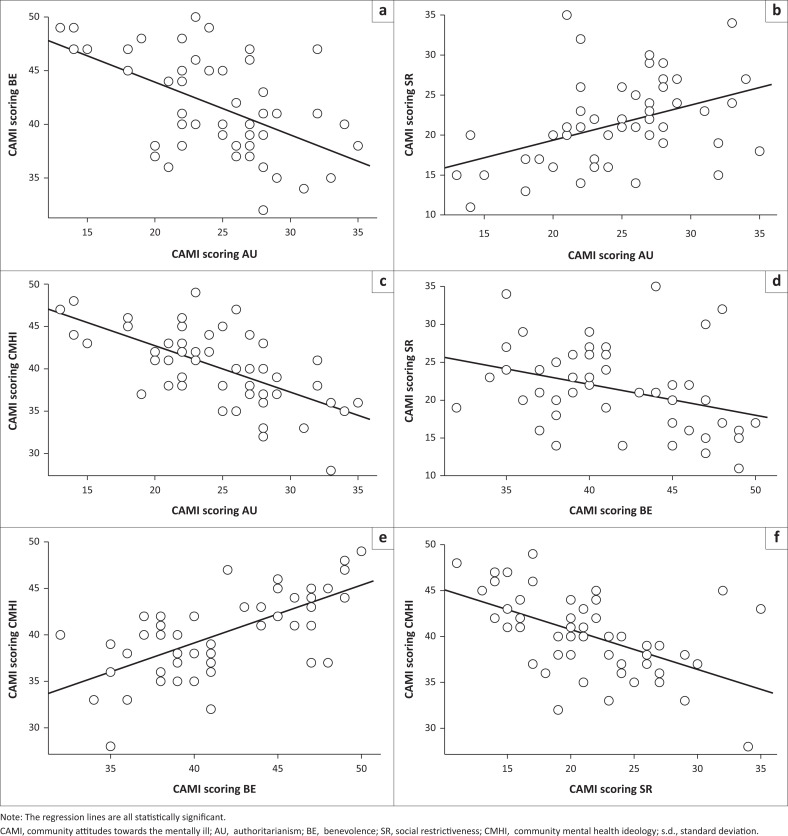
The pairwise relationship between the four Community Attitudes Towards the Mentally Ill categories: (a) Negative relationship between BE and AU; (b) Positive relationship between SR and AU; (c) Negative relationship between CMHI and AU; (d) Negative relationship between SR and BE; (e) Positive relationship between CMHI and BE; (f) Negative relationship between CMHI and SR.

The associations between the CAMI categories were all significant and showed four negative and two positive relationships (Figure 1). The relationship between AU and BE was significantly negative (*R* = 0.55, *p* < 0.001), between AU and SR was significantly positive (*R* = 0.41, *p* = 0.002) and between AU and CMHI was significantly negative (*R* = 0.64, *p* < 0.001). The relationship between BE and SR was significantly negative (*R* = 0.35, *p* = 0.013), between BE and CMHI was significantly positive (*R* = 0.66, *p* < 0.001) and between SR and CMHI was significantly negative (*R* = 0.54, *p* < 0.001). These relationships broadly support the mean values reported earlier in the text but also indicate that individual churchgoers had consistent responses for each category.

Analysis of the CAMI scores revealed no significant differences between congregants (C) and leaders (L) across all four categories, where the mean s.d. scores for congregants and leaders for each category were almost equal (AU C: 24.66 L: 24.82, BE C: 41.72 L: 41.45, SR C: 21.69, L: 21.09, CMHI C: 40.34 L: 39.86) (Table 2). This indicates comparable attitudes towards mental illness regardless of leadership status within the church community.

**TABLE 2:** Mean (s.d.) scores for congregants and leaders for each Community Attitudes Towards the Mentally Ill category.

Congregant or leader	Mean	s.d.	Statistics		
			*t*	*df*	*p*
**CAMI scoring AU**	-	-	−0.12	47.87	0.908
Congregant	24.66	6.05	-	-	-
Leader	24.82	3.89	-	-	-
**CAMI scoring BE**	-	-	0.20	43.63	0.842
Congregant	41.72	4.55	-	-	-
Leader	41.45	4.88	-	-	-
**CAMI scoring SR**	-	-	0.40	48.64	0.689
Congregant	21.69	6.27	-	-	-
Leader	21.09	4.33	-	-	-
**CAMI scoring CMHI**	-	-	0.39	48.77	0.697
Congregant	40.34	4.85	-	-	-
Leader	39.86	3.92	-	-	-

Note: Statistics = Welch’s t-tests.

CAMI, community attitudes towards the mentally ill; AU, authoritarianism; BE, benevolence; SR, social restrictiveness; CMHI, community mental health ideology; s.d., standard deviation.

Significant differences were observed concerning personal history of mental illness and BE and CMHI scores (Table 3). Participants with a personal history of mental illness exhibited higher mean scores in both BE and CMHI compared to those without such a history, indicating more positive attitudes towards mental illness among individuals with lived experiences.

**TABLE 3:** Mean (s.d.) scores of having (Yes) or not (No) a personal history of mental illness for each Community Attitudes Towards the Mentally Ill category.

Personal history of mental illness	Mean	s.d.	Statistics		
			*t*	*df*	*p*
**CAMI scoring AU**	-	-	1.82	19.39	0.084
Yes	22.40	6.45	-	-	-
No	25.69	4.29	-	-	-
**CAMI Scoring BE**	-	-	−2.17†	27.79†	0.039†
Yes	43.67	4.30	-	-	-
No	40.75	4.57	-	-	-
CAMI Scoring SR	-	-	1.98	24.26	0.059
Yes	19.07	5.65	-	-	-
No	22.42	5.16	-	-	-
**CAMI scoring CMHI**	-	-	−2.78†	27.66†	0.010†
Yes	42.60	4.01	-	-	-
No	39.11	4.24	-	-	-

Note: Statistics = Welch’s t-tests.

CAMI, community attitudes towards the mentally ill; AU, authoritarianism; BE, benevolence; SR, social restrictiveness; CMHI, community mental health ideology; s.d., standard deviation.

† , Significant outcomes.

In contrast, no significant differences emerged between participants who knew someone with mental illness and those who did not across all CAMI categories (Table 4). This suggests that familiarity with individuals affected by mental illness did not notably influence attitudes towards mental health within the sampled population.

**TABLE 4:** Mean (s.d.) scores of knowing (Yes) or not (No) someone with mental illness for each Community Attitudes Towards the Mentally Ill category.

Knowing someone with a mental illness	Mean	s.d.	Statistics		
			*t*	*df*	*p*
**CAMI scoring AU**	-	-	1.59	30.63	0.123
Yes	26.33	6.45	-	-	-
No	24.23	4.29	-	-	-
**CAMI scoring BE**	-	-	−0.92	29.17	0.367
Yes	40.75	4.30	-	-	-
No	41.87	4.57	-	-	-
**CAMI scoring SR**	-	-	0.77	18.41	0.449
Yes	22.50	5.65	-	-	-
No	21.10	5.16	-	-	-
**CAMI scoring CMHI**	-	-	−0.06	30.40	0.952
Yes	40.08	4.01	-	-	-
No	40.15	4.24	-	-	-

Note: Statistics = Welch’s t-tests.

CAMI, community attitudes towards the mentally ill; AU, authoritarianism; BE, benevolence; SR, social restrictiveness; CMHI, community mental health ideology; s.d., standard deviation.

## Discussion

This study investigated Christian leaders’ and congregants’ attitudes towards mental illness using the CAMI questionnaire. The study sample consisted of 51 participants, including 29 congregants and 22 leaders. The majority of the participants were female (80.4%), with 19.6% being male, were aged between 25 years and 35 years old (58.8%) and had a tertiary-level education (82.4%). These characteristics align with previous studies on the Christian population which often included participants who were predominantly female, aged between 18 years and 35 years and possessed tertiary-level education.^23,30^ The gender disparity can be explained by the fact that women have been found to attend church more frequently than men.^12^ Previous studies have shown that individuals with a higher level of education tend to participate more in surveys, have more knowledge about mental health issues and are more likely to recognise the importance of addressing them.^31,32^ The majority of the participants were young adults and this could have been because younger adults are becoming more aware of mental health and are likely to engage in mental health discussions and research.^33^

The participants in the current study mostly belonged to the Pentecostal or Charismatic denomination (52.9%), whereas they represent only 7.3% of the South African Christian population. Only 5.9% belonged to the African Independent denomination, the largest South African group (40.8%). The distribution of all the denominations in this study varied greatly from the national religious demographic distribution.^12^ Several factors influenced this distribution. Participation bias may have led individuals from certain denominations to be more likely to participate. Additionally, the administrative structures of the denominations varied, impacting participation rates and responsiveness. The implications of this level of participation suggest that the study’s findings may be limited in their ability to reflect the broader attitudes of the South African Christian population.

The interpretation of CAMI scores showed low AU and SR scores, alongside high BE and CMHI scores, indicative of positive attitudes towards mental illness. This pattern suggests a prevailing inclination towards empathy, support and a community-based approach to mental health issues within the sampled population. These findings are consistent with research showing that women, who made up a large portion of the sample, generally exhibit greater empathy.^34^ Additionally, about three-quarters of participants had prior experience with someone who had a mental illness which is shown to reduce stigmatising attitudes towards individuals with mental illness.^20^ These findings align with broader trends observed in studies examining attitudes towards mental illness among religious communities albeit with notable variations across different cultural and religious contexts.^22,23,30^

Previous research examining the attitudes of Christians towards mental illness has predominantly focused on Christian leaders, who were mostly male and over the age of 40. The findings from these studies have been mixed, with some indicating positive attitudes and others revealing negative perceptions.^13,20,21,22^ In the current study, most leaders were between the ages of 25 and 35 and exhibited a more positive attitude towards mental illness, with high scores in BE and CMHI and lower scores in AU and SR. This finding is similar to a 2023 South African study of Pentecostal congregants, which found that the predominantly female participants with higher education level had less stigmatising attitudes towards mental illness.^24^ The age and education level of the majority of participants may have positively influenced their attitudes as they could have been exposed to more mental health advocacy and education.

Of particular interest is the comparison between Christian leaders and congregants. No statistically significant differences were observed in the mean scores across the four CAMI categories between these two groups. This finding challenges conventional assumptions regarding the potential influence of leadership roles within religious communities on attitudes towards mental illness.^35^ As congregants look to church leaders for support and assistance when going through difficult times, including emotional and mental distress, one would then expect church leaders to have less stigmatising attitudes towards mental illness, in comparison with congregants.

Individuals with a personal history of mental illness exhibited significantly higher scores in BE and CMHI and lower scores in AU and SR, indicative of reduced stigma compared to those without such a history. This finding, which is in keeping with other studies,^36,37^ highlights the potential for empathy and understanding to emerge from lived experiences. Those who have experienced mental illness themselves are often less likely to hold stigmatising beliefs about others with mental health conditions.

Acquaintance with individuals affected by mental illness did not appear to influence attitudes towards mental illness among participants, as they had scores similar to individuals who did not know someone with a mental illness. Some research, including this study, indicate that simply knowing someone with a mental illness does not always translate into more positive attitudes.^38,39^ Conversely, other studies have suggested that familiarity with individuals affected by mental illness may yield a beneficial impact, enhancing understanding and fostering empathetic attitudes towards mental health challenges.^40,41^

The results indicate generally positive views towards mental health, with low levels of AU and SR and higher levels of BE and CMHI. However, the varying levels of engagement across denominations, potentially influenced by administrative processes, may suggest that factors like stigma or denominational differences could play a role in shaping responses. This raises important questions about how organisational and cultural factors within different denominations may impact attitudes towards mental health.

### Limitations

Notable limitations of the study are the small sample size and denomination distribution. The imbalance in the denomination distribution made it challenging to ensure that the findings were representative of the diverse religious landscape of the country. Additionally, navigating the bureaucratic processes within different denominations proved complex as each had its unique administrative requirements and sensitivities. These challenges required careful consideration to respect the distinct organisational structures. Consequently, the researcher had to rely on community members to distribute the questionnaires, which may have introduced selection bias and limited the diversity of the sample. This approach could have affected the representativeness of the results, impacting their generalisability. This was a cross-sectional design and did not allow for causal reasoning. The use of a convenience sample further constrained the generalisability of findings as the people who responded may be more likely to have knowledge of, or a more positive attitude towards, mental illness. Other limitations were that a self-report questionnaire was administered which may bias results through reduced self-awareness and the need for social desirability.

## Conclusion

The Christian community plays a significant role in supporting mental health, as the sense of belonging and social support within these communities is associated with better mental health outcomes.^9,42,43^ Encouraging acceptance and understanding may help reduce the stigma surrounding mental illness, creating a safe environment for individuals to seek help.^43^ Research suggests that fostering mutual understanding between mental healthcare providers and clergy can enhance the quality of care and outcomes.^44^ The findings in this study offer some insights into Christian leaders’ and congregants’ attitudes towards mental illness in SA, underscoring the need for further research to understand these complex dynamics within the religious community. The study also highlights the challenges of engaging the Christian community, particularly the disproportionate participation across denominations. Future research should prioritise building trust with a broader range of denominations and developing trust with church leadership to improve participation rates. Interdisciplinary training programmes that equip mental healthcare workers with cultural competence and sensitivity to religious beliefs can strengthen relations with the Christian community through workshops and joint community initiatives, leading to more holistic and culturally responsive mental health services.

## References

[1] Whiteford HA, Degenhardt L, Rehm J, et al. Global burden of disease attributable to mental and substance use disorders: Findings from the Global Burden of Disease Study 2010. Lancet. 2013;382(9904):1575–1586. 10.1016/S0140-6736(13)61611-6 23993280

[2] Dell’Osso B, Glick ID, Baldwin DS, Altamura AC. Can long-term outcomes be improved by shortening the duration of untreated illness in psychiatric disorders? A conceptual framework. Psychopathology. 2013;46(1):14–21. 10.1159/000338608 22890286

[3] Memon A, Taylor K, Mohebati LM, et al. Perceived barriers to accessing mental health services among black and minority ethnic (BME) communities: A qualitative study in Southeast England. BMJ Open. 2016;6(11):e012337. 10.1136/bmjopen-2016-012337 PMC5128839 27852712

[4] Olivier J, Tsimpo C, Gemignani R, et al. Understanding the roles of faith-based health-care providers in Africa: Review of the evidence with a focus on magnitude, reach, cost, and satisfaction. Lancet. 2015;386(10005):1765–1775. 10.1016/S0140-6736(15)60251-3 26159398

[5] Weich S, Lewis G. Income inequalities and prevalence of common mental disorders in Britain. Br J Psychiatry. 2001;178(3):222–227. 10.1192/bjp.178.3.222 11230032

[6] Patel V, Kleinman A. Poverty and common mental disorders in developing countries. Bull World Health Organ. 2003;81(8):609–615. 14576893 PMC2572527

[7] Marwaha S, Johnson S. Schizophrenia and employment. Soc Psychiatry Psychiatr Epidemiol. 2004;39(5):337–349. 10.1007/s00127-004-0762-4 15133589

[8] Ribeiro WS, Andreoli SB, Ferri CP, Prince M, Mari JJ. Exposure to violence and mental health problems in low and middle-income countries: A literature review. Brazilian J Psychiatry. 2009;31(2):S49–S57. 10.1590/S1516-44462009000600003 19967200

[9] Chalfant HP, Heller PL, Roberts A, Briones D, Aguirre-Hochbaum S, Farr W. The clergy as a resource for those encountering psychological distress. Rev Relig Res. 1990;31(3):305. 10.2307/3511620

[10] Karl Peltzer. Faith healing for mental disorders in the Northern Province (South Africa). J Relig Afr. 1999;24:387–402. 10.1163/157006699X00395

[11] Wood E, Watson R, Hayter M. To what extent are the Christian clergy acting as frontline mental health workers? A study from the North of England. Ment Health Relig Cult. 2011;14(8):769–783. 10.1163/157006699X00395

[12] Schoeman WJ. South African religious demography: The 2013 General Household Survey. HTS Teologiese Stud/Theol Stud. 2017;73(2):7. 10.4102/hts.v73i2.3837

[13] Yamada AM, Lee KK, Kim MA, Moine M, Oh H. Beliefs about etiology and treatment of mental illness among Korean Presbyterian Pastors. J Relig Health. 2019;58(3):870–880. 10.1007/s10943-018-0720-1 30341709

[14] Stanford MS. Demon or disorder: A survey of attitudes toward mental illness in the Christian church. Ment Health Relig Cult. 2007;10(5):445–449. 10.1080/13674670600903049

[15] Altmann TK. Attitude: A concept analysis. Nursing Forum. 2008;43(3):144–150. 10.1111/j.1744-6198.2008.00106.x 18715347

[16] Hugo CJ, Boshoff DEL, Traut A, Zungu-Dirwayi N, Stein DJ. Community attitudes toward and knowledge of mental illness in South Africa. Soc Psychiatry Psychiatr Epidemiol. 2003;38(12):715–719. 10.1007/s00127-003-0695-3 14689176

[17] Girma E, Tesfaye M, Froeschl G, Möller-Leimkühler AM, Müller N, Dehning S. Public stigma against people with mental illness in the Gilgel Gibe Field Research Center (GGFRC) in Southwest Ethiopia. Preux PM, editor>. PLoS One. 2013;8(12):e82116. 10.1371/journal.pone.0082116 24324756 PMC3853185

[18] Reta Y, Tesfaye M, Girma E, Dehning S, Adorjan K. Public stigma against people with mental illness in Jimma Town, Southwest Ethiopia. van Amelsvoort T, editor. PLoS One. 2016;11(11):e0163103. 10.1371/journal.pone.0163103 27893745 PMC5125563

[19] Ukpong DI, Abasiubong F. Stigmatising attitudes towards the mentally ill: A survey in a Nigerian university teaching hospital. S Afr J Psychiatry. 2010;16(2):5. 10.4102/sajpsychiatry.v16i2.238

[20] Aramouny C, Kerbage H, Richa N, Rouhana P, Richa S. Knowledge, Attitudes, and beliefs of Catholic Clerics’ regarding mental health in Lebanon. J Relig Health. 2020;59(1):257–276. 10.1007/s10943-019-00758-1 30661138

[21] Igbinomwanhia N, James B, Omoaregba J. The attitudes of clergy in Benin City, Nigeria towards persons with mental illness. Afr J Psychiatry. 2013;16(3):196–200. 10.4314/ajpsy.v16i3.26 23739822

[22] Stanford M, Philpott D. Baptist senior pastors’ knowledge and perceptions of mental illness. Ment Health Relig Cult. 2011;14(3):281–290. 10.1080/13674670903511135

[23] Salifu Yendork J, Brew GB, Sarfo EA, Kpobi L. Mental illness has multiple causes: Beliefs on causes of mental illness by congregants of selected neo-prophetic churches in Ghana. Ment Health Relig Cult. 2018;21(7):647–666. 10.1080/13674676.2018.1511694

[24] Hlongwane N, Juby V. Knowledge, attitudes, and help-seeking behaviour for mental illness in a Christian community. S Afr J Psychiatry. 2023;29:a2139. 10.4102/sajpsychiatry.v29i0.2139 PMC10696576 38059199

[25] Nhlumayo, Lethukuthula Nkanyiso. Church leaders’ understandings of how Christian beliefs inform mental illness identification and remediation in effected members: A scoping review [homepage on the Internet]. Department of Psychology, University of Kwazulu-Natal; 2021 [cited 2023 Mar 03]. Available from: https://researchspace.ukzn.ac.za/handle/10413/20723

[26] Stanford MS, McAlister KR. Perceptions of serious mental illness in the local church. J Relig Disabi Health. 2008;12(2):144–153. 10.1080/15228960802160654

[27] Taylor SM, Dear MJ. Scaling community attitudes toward the mentally ill. Schizophr Bull. 1981;7(2):225–240. 10.1093/schbul/7.2.225 7280561

[28] Hinkelman L, Granello DH. Biological sex, adherence to traditional gender roles, and attitudes towards persons with mental illness: An exploratory investigation. J Ment Health Counsel. 2003;25(4):259–270. 10.17744/mehc.25.4.tglx0uudjk7q5dpk

[29] CAMI Scale [homepage on the Internet]. [cited 2021 Oct 11]. Available from: https://camiscale.com/cami-questionnaire/

[30] Gray AJ. Attitudes of the public to mental health: A church congregation. Ment Health Relig Cult. 2001;4(1):71–79. 10.1080/713685617

[31] Pescosolido BA, Martin JK, Long JS, Medina TR, Phelan JC, Link BG. “A Disease Like Any Other”? A decade of change in public reactions to schizophrenia, depression, and alcohol dependence. Am J Psychiatry. 2010;167(11):1321–1330. 10.1176/appi.ajp.2010.09121743 20843872 PMC4429867

[32] Jorm AF. Mental health literacy: Public knowledge and beliefs about mental disorders. Br J Psychiatry. 2000;177(5):396–401. 10.1192/bjp.177.5.396 11059991

[33] Gulliver A, Griffiths KM, Christensen H. Perceived barriers and facilitators to mental health help-seeking in young people: A systematic review. BMC Psychiatry. 2010;10(1):113. 10.1186/1471-244X-10-113 21192795 PMC3022639

[34] Toussaint L, Webb JR. Gender differences in the relationship between empathy and forgiveness. J Soc Psychol. 2005;145(6):673–685. 10.3200/SOCP.145.6.673-686 16334893 PMC1963313

[35] Campbell AD. Clergy perceptions of mental illness and confronting stigma in congregations. Religions. 2021;12(12):1110. 10.3390/rel12121110

[36] Couture S, Penn D. Interpersonal contact and the stigma of mental illness: A review of the literature. J Ment Health. 2003;12(3):291–305. 10.1080/09638231000118276

[37] Alexander L, Link B. The impact of contact on stigmatizing attitudes toward people with mental illness. J Ment Health. 2003;12(3):271–289. 10.1080/0963823031000118267

[38] Corrigan PW, Watson AC. Understanding the impact of stigma on people with mental illness. World Psychiatry. 2002;1(1):16–20. 16946807 PMC1489832

[39] Crisp AH, Gelder MG, Rix S, Meltzer HI, Rowlands OJ. Stigmatisation of people with mental illnesses. Br J Psychiatry. 2000;177(1):4–7. 10.1192/bjp.177.1.4 10945080

[40] Evans-Lacko S, Henderson C, Thornicroft G. Public knowledge, attitudes and behaviour regarding people with mental illness in England 2009–2012. Br J Psychiatry. 2013;202(s55):s51–S57. 10.1192/bjp.bp.112.112979 23553695

[41] Abi Doumit C, Haddad C, Sacre H, et al. Knowledge, attitude and behaviors towards patients with mental illness: Results from a national Lebanese study. Vaingankar JA, editor. PLoS One. 2019;14(9):e0222172. 10.1371/journal.pone.0222172 31525219 PMC6746362

[42] Koenig HG. Religion, spirituality, and health: The research and clinical implications. ISRN Psychiatry. 2012;2012:1–33. 10.5402/2012/278730 PMC3671693 23762764

[43] Wang J, Mann F, Lloyd-Evans B, Ma R, Johnson S. Associations between loneliness and perceived social support and outcomes of mental health problems: A systematic review. BMC Psychiatry. 2018;18(1):156. 10.1186/s12888-018-1736-5 29843662 PMC5975705

[44] Kennedy G, Kennedy GA, Macnab FA, Ross JJ. The effectiveness of spiritual/religious interventions in psychotherapy and counselling: A review of the recent literature. Melbourne: PACFA; 2015.

